# Systemic and anti-nociceptive effects of prolonged lidocaine, ketamine, and butorphanol infusions alone and in combination in healthy horses

**DOI:** 10.1186/1746-6148-10-S1-S6

**Published:** 2014-07-07

**Authors:** Johanna R Elfenbein, Sheilah A Robertson, Robert J MacKay, Butch KuKanich, L Chris Sanchez

**Affiliations:** 1 Island Whirl Equine Colic Research Laboratory, Department of Large Animal Clinical Sciences, University of Florida College of Veterinary Medicine, Gainesville, FL, USA; 2 Department of Anatomy & Physiology, Kansas State University, Manhattan, KS, USA; 3 Department of Veterinary Pathobiology, Texas A&M University College of Veterinary Medicine & Biomedical Sciences, College Station, TX, USA; 4 Department of Small Animal Clinical Sciences, Michigan State University College of Veterinary Medicine, East Lansing, MI, USA

## Abstract

**Background:**

Prolonged drug infusions are used to treat horses with severe signs of pain, but can be associated with altered gastrointestinal transit. The purpose of this study was to determine the effects of prolonged constant rate infusions (CRI) of lidocaine (L), butorphanol (B), and ketamine (K) alone and in combination on gastrointestinal transit, behavior, and thermal nociceptive threshold in healthy horses.

**Methods:**

Eight healthy adult horses were used in a randomized, cross-over, blinded, prospective experimental trial. Interventions were saline, L, K, B, LK, LB, BK, and LBK as an intravenous CRI for 96 hours. Drugs were mixed or diluted in saline; following a bolus, CRI rate was 0.15mL/kg/hr with drug doses as follows: L – 1.3 mg/kg then 3 mg/kg/hr; B – 0.018 mg/kg then 0.013 mg/kg/hr; K – 0.55 mg/kg then 0.5 mg/kg/hr. Two-hundred plastic beads were administered intragastrically by nasogastric tube immediately prior to the bolus. Feces were collected every 2 hours, weighed, and beads manually retrieved. Behavior was scored every 2 hours, vital parameters every 6 hours, and thermal nociceptive threshold every 12 hours for 96 hours. Drug concentrations in the LBK solution were tested every 6 hours for 72 hours.

**Results:**

Four of 64 trials (3 LBK, 1 BK) were discontinued early due to signs of abdominal discomfort. There were no apparent differences between groups in vital parameters or thermal threshold. Transit time was delayed for LB and LBK with a corresponding decrease in fecal weight that was most severe in the final 24 hours of infusion. Significant changes in behavior scores, vital parameters, or thermal threshold were not observed. The concentration of each drug in the combined solution declined by less than 31% over the sampling period.

**Conclusions:**

Drug combinations containing butorphanol cause an apparent delay in gastrointestinal transit in healthy horses without substantially affecting somatic nociception at the doses studied. Combinations of lidocaine and ketamine may have less impact on gastrointestinal transit than infusions combined with butorphanol. Further work is needed to determine the effects of these drugs in painful or critically ill patients.

## Background

Identification and alleviation of pain is essential for welfare of all species. The most commonly used analgesic medications in horses include the alpha_2_-adrenergic agonists, non-steroidal anti-inflammatory drugs (NSAIDs), and opioids. Despite well-documented analgesic properties, the alpha_2_-adrenergic agonists profoundly decrease gastrointestinal motility [1-3] making these drugs a poor choice for prolonged administration. NSAIDs can lead to gastric and colonic ulceration and renal tubular necrosis, potentially life-threatening side effects. Severe pain may be refractory to single analgesic therapy; thus its management may require a multimodal approach, employing drugs with different mechanisms of action. Despite the potential for improved analgesia [4], use of drug combinations may also increase the potential for adverse effects, especially alterations in gastrointestinal motility or behavior [2] when drugs are combined at the dosages used for monotherapy.

Lidocaine, a local anesthetic, is commonly administered as a constant rate infusion (CRI) in horses following exploratory laparotomy for its potential analgesic and prokinetic properties [5-10]. Ketamine, an N-methyl-D-aspartate (NMDA) receptor antagonist, has anti-nociceptive properties when administered as a CRI at sub-anesthetic doses in both anesthetized and conscious horses [11,12]. The opioid drug butorphanol, a kappa (OP2) agonist and competitive mu (OP3) antagonist, is commonly administered in the horse with varying success. No anti-nociceptive properties were noted in clinically normal horses in response to visceral distension or noxious thermal stimulus [13] but pain scores were significantly decreased in horses administered butorphanol as a constant rate infusion in the immediate post-operative period following exploratory laparotomy [14].

Overall gastrointestinal transit time from the ingestion of a meal to passage of feces is one of the most clinically applicable objective evaluations of gastrointestinal function as it incorporates all segments of the gastrointestinal tract. It is commonly measured by passage of either liquid or particulate markers administered via nasogastric tube, which are then either physically recovered or identified radiographically in feces [15,16]. Clinical methods for assessing gastrointestinal motility include auscultation of gastrointestinal borborygmi and passage of feces. In this study we combined objective measures of total gastrointestinal transit time and fecal output and subjective assessment of borborygmi scores for a global assessment of gastrointestinal motility.

Thermal threshold (TT) testing has been successfully used in adult horses and foals to evaluate somatic nociception [10,17,18]. A wireless TT system allows testing to be performed with the horse unrestrained, [17] which allows the horse to display the full range of behavioral responses to a noxious stimulus and reduces the potential effects of restraint and presence of an investigator on the response. Thermal nociceptive threshold testing is one measure of nociception. The benefits of this mode of testing are that it is repeatable and causes no tissue damage thus reducing the animal welfare concerns with repeated nociceptive testing.

The purpose of the study reported here was to evaluate the effects of lidocaine, ketamine, and butorphanol, alone and in combination, on total gastrointestinal transit time, somatic nociception, and behavior in clinically normal horses. From an immediate clinical standpoint, this study provides clinicians already using these drugs in combination with knowledge of both the anti-nociceptive and systemic effects of the medications in healthy, non-painful, horses.

## Methods

### Animals

Eight Thoroughbred or Thoroughbred-cross horses (5 geldings, 3 mares; aged 5-20 years; weight 463-558 kg) were used. Each horse was instrumented with a permanent gastric cannula placed for the purpose of other experiments. Mares were used during behavioral diestrus. There was at least 1 week between the end of one study period and the beginning of the next, during which horses were maintained on grass pasture with concentrate and grass hay available. All experimental procedures were approved by the University of Florida Institutional Animal Care and Use Committee.

Horses were stalled 12-18 hours prior to each study period for acclimation. Horses were studied in groups of 2-3 to reduce the effects of social isolation on study outcome. Body weight was measured following acclimation and at the end of each study. Horses were offered free choice grass hay (in pre-weighed bins) and water (in graduated buckets) and twice-daily concentrate during the course of the study. Hay and water consumption was measured and recorded every 4 hours throughout the study period.

### Treatments

Each study period began between 0800 and 1000 hours. Each horse received each treatment in an orthogonal Latin square design; investigators were blinded as to treatment. Treatments were 0.9% sodium chloride (Placebo, P; 60 mL bolus, 0.15 mL/kg/hr), lidocaine hydrochloride (Lidocaine hydrochloride injectable-2%, Phoenix Pharmaceutical, Inc., St. Joseph, MO, USA) (L; 1.3 mg/kg bolus, 3 mg/kg/hr), ketamine hydrochloride (KetaVed^®^, Vedco, St. Joseph, MO, USA) (K; 0.55 mg/kg bolus, 0.5 mg/kg/hr), butorphanol tartrate (Torbugesic^®^, Fort Dodge Animal Health, Fort Dodge, IA, USA) (B; 0.018 mg/kg bolus, 0.013 mg/kg/hr) administered individually and in combination (P, L, B, K, LK, LB, KB, and LBK) for a total of 8 treatments. No diluent was needed for treatments including lidocaine; all other drugs and combinations were diluted in sodium chloride. Infusions were prepared in 1-L increments. The bolus was administered over 15 minutes and the CRI administration began immediately following the bolus with a computerized infusion pump for 96 hours.

### Drug stability

Prior to the live animal study, stability of the drugs in combination was assessed. Ketamine and butorphanol were added to lidocaine (100 mL total volume) at the dosage indicated for LBK infusion. The drug combination was maintained inside in a climate controlled environment at room temperature (approximately 20-22ºC) in ambient light. A 2mL sample was removed and frozen at -20ºC every 6 hours for 72 hours. The concentrations of butorphanol, lidocaine and ketamine were determined by liquid chromatography (Shimadzu Prominence, Shimadzu Scientific Instruments, Columbia, MD, USA) and mass spectrometry (API 2000, Applied Biosystems, Foster City, CA, USA). The internal standards were fentanyl (Cerilliant Corporation, Round Rock, TX, USA), mepivacaine (Sigma-Aldrich, St Louis, MO, USA), and ketamine d4 (Cerilliant Corporation, Round Rock, TX, USA) for butorphanol, lidocaine and ketamine respectively. The qualifying and quantifying ions for butorphanol, lidocaine and ketamine were mass to charge ratio (*m/z*) 328.21→157.2, 235.22→86.2, and 238.09→124.9, respectively. The qualifying and quantifying ions for fentanyl, mepivacaine and ketamine d4 were (*m/z*) 337.14→105.3, 247.21→98.2, and 242.16→129.1, respectively. Standard curves for each of the analytes were accepted if they were linear with calculated concentrations within 15% of the actual concentration and the correlation coefficient was at least 0.99. The standards and the tested solutions were diluted in 0.1% formic acid in water. The mobile phase consisted of A: acetonitrile and B: 0.1% formic acid in deionized water at a flow rate of 0.4 mL/min. The mobile phase started at 90% B with a linear gradient to 40% B at 4 minutes and back to 90% B at 5 minutes with a total run time of 6.5 minutes. Separation was achieved with a C18 column (ACE C18AR, 150 mm x 3.0 mm x 5 μm) (MAC-MOD Analytical, Chadds Ford, PA, USA) maintained at 40°C.

### Data collection

For each trial, a 14-g intravenous catheter was placed aseptically into each jugular vein and horses were fitted with a fecal collection device. A nasogastric tube was placed and 200 3x5-mm plastic beads administered in 1 L water by gravity flow using a funnel. Different color beads were used for each trial to avoid any carryover from previous trials. After bead administration was complete, treatment bolus then CRI began. Vital signs (heart rate, respiratory rate, and rectal temperature), behavior scores, and gastrointestinal borborygmi scores were recorded as previously described [19] every 6 hours for the study duration. Feces were collected, weighed, and beads manually retrieved every 2 hours. Following retrieval of 180 beads, the fecal collection device was removed and feces collected from the stall floor were weighed every 2 hours. Hay (kg) and water (L) consumption was recorded every 6 hours.

Thermal threshold (TT) testing was performed using a wireless device as previously described [17]. Briefly, an area on one side of the withers (alternating randomly) was shaved and the TT probe placed in direct contact with the skin. The probe was secured using an adjustable nylon strap around the thorax. Consistent pressure between the probe and skin was ensured by inflating a modified blood pressure cuff and pressure monitored by a sensor within the device. Skin temperature was recorded following an equilibration period of at least 5 minutes. The device was activated by a wireless hand-held toggle switch by an investigator positioned outside of the horse’s stall. The device was activated when the horse was not visibly interacting with the investigator. Heating was discontinued either when the horse displayed a response or at 55ºC, whichever occurred first. Responses included a skin twitch, looking at the flank, or an abrupt lifting of the head. TT was performed twice prior to treatment bolus (baseline), 15 minutes into the drug infusion then every 12 hours for the duration of the study. TT was not performed on one horse due to its failure to respond to the stimulus at the baseline measurement. For time points reaching the cut-off value (55ºC), responses were recorded as 55.5 for data analysis.

### Statistical analysis

Unless indicated otherwise, all data are expressed as least square mean ± standard error of the mean. For TT, baseline was considered the mean of two measurements. The data for each response variable conformed to a split-plot ANOVA according to the model: Y = Treatment + Period + Horse + Error_1_ + Time + Treatment*Time + Error_2_. When period was not a significant factor for a given response variable, period was eliminated from each model and data were then analyzed by a three-factor ANOVA with the fixed factors of Time and Treatment and the random factor of Horse. When significant main effects or interactions occurred, post hoc comparisons were made by the Bonferroni t test. A commercial software package (SAS/STAT, SAS Institute Inc., Cary, NC, USA) was used for all analyses and a *p* <0.05 was considered significant. For post hoc comparisons, the critical *p* was considered 0.05/number of comparisons.

Mean fecal bead passage fit to a four-parameter logistic equation (SigmaPlot 11.0, Systat Software, Inc., San Jose, CA, USA) that was then used to calculate the time to passage of 1, 25, 50, and 75% of beads for each treatment via regression.

## Results

The infusion was discontinued for 3 horses in the LBK group (after a mean of 38 hours) and 1 in the BK group (after 16 hours) due to signs of colic; their data were excluded from analysis. Signs of pain were mild in all horses (flank watching, lying down), none developed nasogastric reflux, and all responded to medical therapy (flunixin meglumine, water and electrolytes via nasogastric tube). No other problems were observed.

The percentage of active drug, relative to time 0, detected in the combined solution (as prepared for all infusions) after 6, 12, 24, 48, and 72 hours of storage was as follows: butorphanol - 78, 92, 81, 83, 79%; ketamine - 72, 90, 75, 74, 69%; lidocaine - 88, 93, 108, 85, 87%. Each 1L bag was administered within 15 hours.

**Figure 1:**
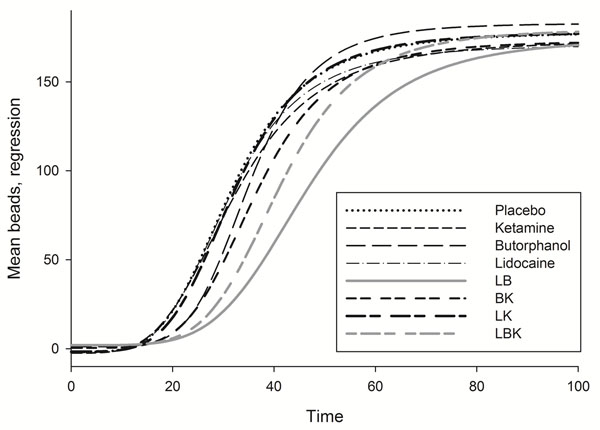
**Four parameter logistic regression equations generated from the mean of beads collected in feces over time after intragastric administration (200 beads).** See Table 1 for key to treatments.

Mean fecal bead passage logistic regression curves are presented in Figure 1; time to passage of 1, 25, 50, and 75% of beads (T_1_, T_25_, T_50_, T_75_) are presented in Table 1. Transit time was significantly delayed in the LB and LBK groups.

**Table 1:** Mean ± SD time (hours) to passage of 1 (T_1_), 25 (T_25_), 50 (T_50_), and 75 (T_75_)% of beads after intragastric administration (200 beads).

	T_1_	T_25_	T_50_	T_75_
P	14.1 ±4.5^a^	27.3 ±5.9^a^	35.1 ±9.1^a^	42.2 ±11.0

L	16.0 ±5.7^a^	29.2 ±9.4^a,c^	38.2 ±17.7^a,b^	41.6 ±12.9

B	15.8 ±5.2^a,b^	31.1 ±6.9^a,b,c^	36.3 ±7.2^a,b^	43.5 ±7.5

K	14.7 ±4.1^a^	28.6 ±8.8^a^	36.7 ±12.3^a,b^	48.7 ±18.8

LB	**24.5 ±9.9^b^**	**41.2 ±13.4^b,c^**	**45.5 ±10.9^b^**	57.3 ±14.3

LK	15.2 ±3.5^a^	28.4 ±7.0^a^	35.1 ±9.0^a^	42.6 ±11.6

BK	21.0 ±10.0^a,b^	33.2 ±10.8^a,b,c^	35.9 ±5.9^a,b^	44.7 ±6.2

LBK	19.5 ±7.6^a,b^	**34.0 ±9.1^b,c^**	40.2 ±11.0^a,b^	52.4 ±20.7

Within a column, different superscripts indicate significant difference between groups. Underlined and bolded font indicates significant difference from placebo. Treatments included infusions of 0.9% saline (Placebo, P), lidocaine (L), butorphanol (B), and ketamine (K); added letters indicates drugs administered in combination (i.e. LB signifies administration of lidocaine and butorphanol in combination).

Overall, horses receiving treatments containing butorphanol had decreased fecal output relative to horses receiving other infusions (Table 2). This was most evident in the LB group within 36-48 hours and in the other butorphanol-containing groups by 72-84 hours.

**Table 2:** Mean (±SD) cumulative fecal weight (kg) passed over time.

Time (hr)								
	0-12	13-24	25-36	37-48	49-60	61-72	73-84	85-96

P	10.9±2.6	20.8±4.9	29.0±6.3^a,b^	38.9±7.5^a^	47.5±10.0^a^	57.2±11.0^a^	65.1±14.6^a^	75.6±17.6^a^

L	8.2±2.8	17.8±6.2	25.5±8.0^a,b^	33.9±10.3^a,b^	40.1±11.6^a,b^	48.0±15.2^a,b^	54.3±17.6^a,b^	62.2±21.6^a,b^

B	6.9±3.5	17.5±6.3	24.6±8.9^a,b^	34.4±11.0^a,b^	40.7±13.0^a,b^	48.3±15.8^a,b^	**52.8±17.7^b,c^**	**60.6±20.8^b,c^**

K	10.3±3.0	21.6±6.9	29.8±9.5^a^	39.7±13.6^a^	48.8±18.0^a^	59.2±22.3^a^	66.3±26.2^a^	75.4±29.9^a^

LB	3.7±2.3	10.9±4.7	16.8±6.3^b^	**24.6±8.0^b^**	**30.2±10.0^b^**	**37.0±12.8^b^**	**41.5±15.0^c^**	**47.6±18.2^c^**

LK	9.3±1.7	20.3±5.3	29.3±7.9^a,b^	39.5±10.6^a^	48.0±14.5^a^	59.0±18.2^a^	66.4±21.8^a^	75.4±23.4^a^

BK	6.9±3.4	16.4±5.8	23.3±8.1^a,b^	31.8±10.5^a,b^	38.6±12.9^a,b^	46.5±16.2^a,b^	52.0±20.2^a,b,c^	**60.2±23.9^b^**

LBK	5.2±3.9	12.7±9.4	18.1±13.8^a,b^	25.2±17.7^a,b^	33.3±22.8^a,b^	39.8±27.6^a,b^	45.4±31.9^a,b,c^	**50.9±36.1^b,c^**

Within a column, different superscripts indicate significant difference between groups. Underlined and bolded font indicates significant difference from placebo. See Table 1 for key to treatments.

**Figure 2:**
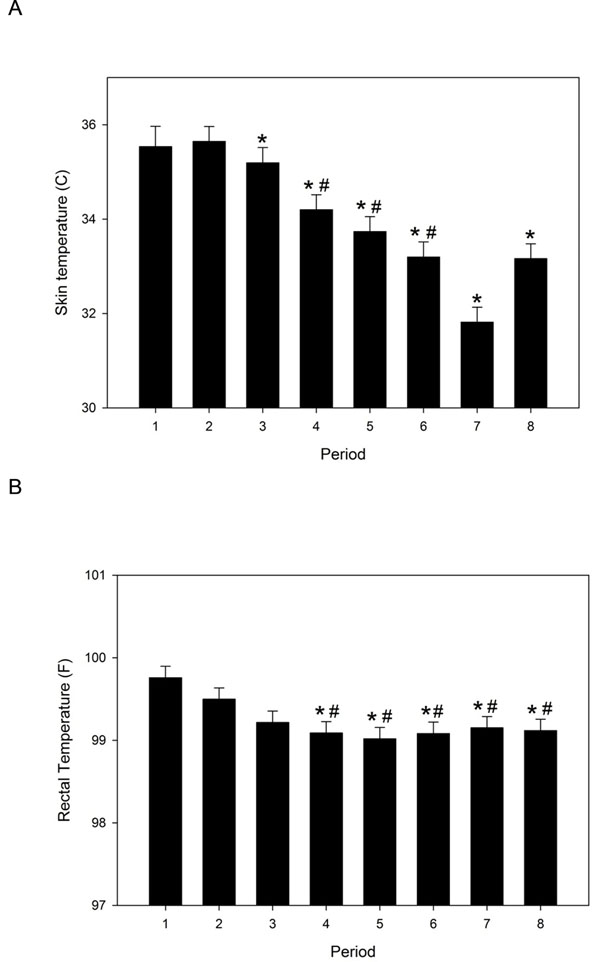
**Least square mean skin (A) and rectal (B) temperatures for each study period.** A study period corresponds to the treatment order for each horse and includes data regardless of treatment administered. The mean start date for study periods 1-8 was as follows: 28 June, 20 July, 15 August, 28 September, 24 October, 17 November, 2 February, and 21 February. Asterisk (*) indicates significant difference from period 1; # indicates significant difference from period 2. Error bars represent SEM.

**Figure 3:**
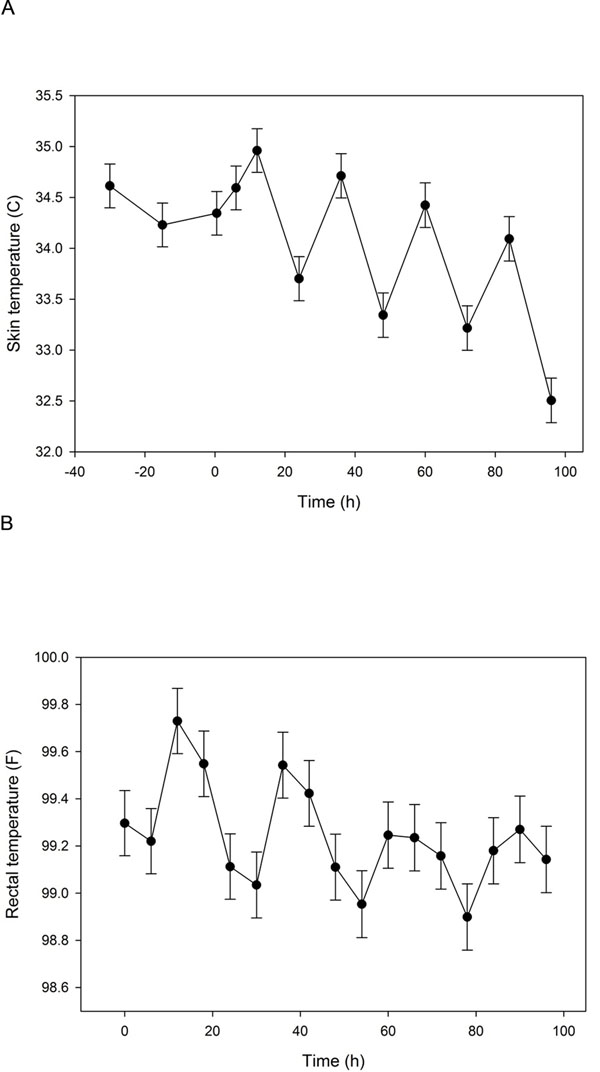
**Least square mean skin (A) and rectal (B) temperatures for each study time.** The study time corresponds to the time after bead administration (time 0) and includes data for all horses and all treatments. See figure 2 for key to indication of statistical significance. Time 0 was between 0800 and 1000 hours. Error bars represent SEM.

There was no significant treatment effect on thermal threshold (Table 3), any vital parameter, behavior or gastrointestinal borborygmi scores, or hay or water consumption (data not shown). There were significant time and/or period effects on skin temperature, rectal temperature, borborygmi scores, heart rate, and body weight. Skin and rectal temperatures were higher in the early experimental periods (the order of experiments, regardless of drug administered) (Figure 2). Body weight was significantly lower in periods 1 and 2 (492.0±9.5 and 494.1±9.4 kg, respectively) relative to periods 4-6 (508.9±9.5, 509.4±9.4, 507.7±9.5 kg). Borborygmi scores were lower in periods 1-3 (7.3±0.6, 7.3±0.6, 6.3±0.6), relative to 5-8 (10.6±0.6, 10.6±0.6, 10.8±0.6, 11.2±0.6). Heart rate was higher in period 1 (41.9±1.2 bpm), relative to periods 5 (37.0±1.2) and 7 (36.9±1.2) and tended to increase then decrease over the 96-hour study period. Skin and rectal temperatures also had a significant time effect, such that temperatures were higher in the afternoon/evening (Figure 3).

**Table 3:** Mean (±SD) thermal threshold (ºC).

Time (hr)												
	-0.5	-0.25	0.5	6	12	24	36	48	60	72	84	96

P	45.7 ±3.4	50.0 ±4.5	52.5 ±3.2	46.4 ±3.9	49.2 ±4.8	51.7 ±4.0	50.8 ±3.8	47.3 ±3.5	51.8 ±3.0	49.7 ±2.3	51.3 ±2.0	52.0 ±4.5

L	47.2 ±1.9	46.9 ±3.0	52.5 ±2.8	49.7 ±3.6	52.2 ±4.1	49.7 ±4.6	50.9 ±2.7	49.2 ±1.4	49.7 ±4.1	51.1 ±3.7	53.2 ±2.5	49.0 ±3.3

B	50.5 ±3.5	49.0 ±3.9	47.9 ±4.2	49.1 ±2.6	52.1 ±2.7	50.8 ±2.7	51.2 ±3.5	50.4 ±3.2	49.1 ±2.5	52.3 ±3.5	53.3 ±2.5	51.1 ±5.2

K	47.0 ±4.1	47.4 ±5.1	48.4 ±5.3	47.8 ±2.4	49.3 ±5.5	50.3 ±2.6	49.7 ±3.4	49.2 ±1.8	52.2 ±3.7	52.1 ±2.8	50.1 ±3.3	49.4 ±4.5

LB	46.4 ±3.8	48.1 ±2.4	53.7 ±2.9	50.1 ±3.8	50.2 ±3.7	51.3 ±2.2	49.8 ±1.0	50.7 ±3.6	49.8 ±3.5	52.0 ±3.2	49.4 ±6.2	52.6 ±2.9

LK	48.7 ±3.0	47.3 ±3.5	53.6 ±2.7	48.1 ±4.3	49.5 ±3.3	49.8 ±3.9	47.6 ±5.6	49.9 ±5.4	49.1 ±4.5	49.7 ±2.4	52.1 ±2.0	49.4 ±3.7

BK	46.2 ±2.6	51.2 ±4.4	50.6 ±3.8	47.5 ±2.8	50.5 ±3.6	48.4 ±3.2	49.7 ±4.8	50.1 ±1.8	50.6 ±3.5	50.7 ±2.8	51.0 ±3.6	51.6 ±3.9

LBK	46.1 ±2.3	45.6 ±5.5	49.7 ±4.4	48.8 ±3.4	51.4 ±3.9	49.1 ±5.0	51.3 ±3.8	49.8 ±1.9	49.2 ±5.0	50.3 ±4.9	48.1 ±5.2	51.6 ±4.9

See Table 1 for key to treatments. No significant period, time, or treatment effects were detected.

The study was conducted from June 2010 through February 2011. Temperature data for the mean date for each study period presented in Table 4.

**Table 4:** Ambient temperature, humidity, and dew point at representative dates for each study period.

Period	Mean start date	High (ºC)	Low (ºC)	Mean humidity (%)	Dew point (ºC)
1	28-Jun 2010	33.3	23.3	75	22.8

2	20-Jul 2010	34.4	24.4	70	23.3

3	15-Aug 2010	33.3	23.9	79	24.4

4	28-Sep 2010	33.3	23.3	79	21.1

5	24-Oct 2010	31.7	12.8	64	14.4

6	17-Nov 2010	24.4	8.3	69	13.9

7	2-Feb 2011	23.9	13.9	75	17.2

8	21-Feb 2011	27.8	11.1	69	13.3

Data represent the daily high and low temperature, mean humidity, and dew point for the mean start date of each study period for Gainesville, FL accessed through http:\\www.wunderground.com.

## Discussion

We report a significant delay in total gastrointestinal transit time and a decrease in cumulative fecal weight as a result of prolonged continuous rate infusion with lidocaine/butorphanol and lidocaine/ketamine/butorphanol combinations. Each drug combination containing butorphanol caused a decrease in cumulative fecal weight during the latter portion of the infusion. The negative effects on cumulative fecal weight do not appear associated with decreased intake, as neither hay nor water consumption was significantly affected.

An infusion time of 96 hours was chosen to ensure that all beads passed during drug infusion. While this duration of infusion may be longer than that commonly used for post-operative patients, horses experiencing severe pain (i.e. laminitis, pleuropneumonia, orthopedic trauma) may require multimodal analgesic therapy of prolonged duration. In fact, we observed significant effects on fecal passage only in the last 24 hours of the 96-hour period with butorphanol, butorphanol/ketamine, and lidocaine/butorphanol/ketamine, whereas there were no substantial effects of these drug combinations (except LBK at T_25_) on bead passage. This suggests that the effects of drug combinations on gastrointestinal motility worsen with longer duration of administration.

A significant treatment effect was not evident for any other measured parameter. Significant period and time effects were observed with rectal temperature, skin temperature and heart rate; a significant period effect was noted with body weight. The changes in skin and rectal temperature appear related to ambient temperature and/or relative humidity, as temperatures were higher in the afternoon/evening hours and during the summer months. These effects were small and likely clinically insignificant, and did not apparently affect treatment or time-treatment interactions because of the orthogonal study design.

Data for the LBK group represent only 5 horses, as the infusion was discontinued in 3 due to colic. Although decreased fecal output and delayed transit were still evident in this group, the negative effects on gastrointestinal function may have been underestimated because the 3 omitted horses likely had the most severely altered gastrointestinal function, resulting in colic. In addition, these horses still displayed signs of abdominal pain despite receiving all three putative analgesic agents together, suggesting either limited visceral analgesia with this drug combination or overriding abdominal pain. Alternatively, it is possible that affected horses experienced some degree of analgesia thus continued to eat hay despite developing ileus, until a more severe problem developed. In prior studies, we were unable to demonstrate visceral anti-nociceptive effects with lidocaine [10] or butorphanol [13] administered as a CRI to healthy horses. It is not known whether anti-nociceptive properties would exist with this drug combination in ill or painful horses.

A significant decrease in cumulative fecal weight for each combination including butorphanol was observed in the current work. We also observed a decrease in fecal weight during the final 24 hours of the 96-hour infusion for horses treated with butorphanol alone, after all of the beads had been passed. This supports prior work in healthy horses [20] and horses post-celiotomy [14], which also showed decreased fecal output. But, in healthy horses, a short duration butorphanol CRI did not significantly alter duodenal motility [13]. The observed decrease in fecal weight and prolonged passage of beads for combinations that included butorphanol may be due to numerous factors including the potential for accumulation of any drug or its metabolites during the infusion, competitive metabolism, or a potential synergistic inhibitory effect on motility of drugs given in combination. Further evaluation of the effects of butorphanol and its metabolites and lidocaine and metabolites on the equine gastrointestinal tract in combination are warranted.

The reported effects of lidocaine administration on gastrointestinal motility in the horse have been variable. Lidocaine administration following exploratory laparotomy has been associated with reduced small intestinal diameter and peritoneal fluid accumulation [8], decreased duration of gastric reflux and hospital stay [7], and reduced incidence of post-operative ileus and improved survival [21]. However, in normal horses lidocaine causes decreased jejunal motility [6] and delayed gastrointestinal transit time after prolonged administration [22]. In this study, we observed no effects of a prolonged lidocaine CRI on gastrointestinal transit, except when combined with butorphanol.

In human and small animal intensive care units, sub-anesthetic doses of ketamine are commonly used for multi-modal pain management and it is believed that ketamine has few adverse systemic effects [23]. Sub-anesthetic doses of ketamine, when administered as constant rate infusions, significantly decreased overall gastrointestinal transit time in comparison with saline control in a prior study [19]. In the study reported here, a lower dose of ketamine was used with no observed adverse effects on behavior scores or total gastrointestinal transit. However, along with the lack of adverse effects, there were no demonstrated anti-nociceptive effects associated with infusions of any drug or combination at dosages used in this study. The lack of antinociceptive effects of ketamine may be due to the lack of central sensitization in these non-painful animals. Further studies in animals with existing pain are warranted and may assess the antinociceptive effects of ketamine infusion more accurately.

## Conclusions

The results of this study, although performed in clinically normal horses, have potential direct clinical application. First, the negative effects of drug combinations on fecal weight began within 36 hours, but apparently worsened with prolonged infusion duration. Second, 3 of the 8 clinically normal horses receiving a combined infusion of lidocaine, butorphanol, and ketamine developed colic. Lidocaine and butorphanol in combination also resulted in a severe delay in gastrointestinal transit and reduced fecal weight, but not colic. Finally, despite the observed delay in gastrointestinal transit, there was no apparent effect on gastrointestinal borborygmi scores. This highlights the poor sensitivity of gastrointestinal borborygmi as a sole clinical marker of gastrointestinal motility. These results are consistent with those of a previous study that showed poor agreement of auscultation scores with electrointestinography [24]. Thus, clinicians using lidocaine, butorphanol, and ketamine in combination should carefully monitor fecal output and be aware of the potential for colic in these patients. The reader should also note that the doses studied likely represent the maximal dosage used, and the effects upon gastrointestinal motility may be limited if lower dosages are used in combination. The lack of observed thermal threshold effects likely represent a lack of drug effect on thermal nociception at doses used in this study. Thermal nociceptive threshold testing is only one measure of nociception and may poorly recapitulate severe clinical pain. Further work is required to determine the effects of these drugs in combination on nociception and gastrointestinal motility in critically ill and painful horses.

## List of abbreviations used

CRI: constant rate infusion; L: lidocaine; B: butorphanol; K: ketamine; LB: lidocaine and butorphanol; LK: lidocaine and ketamine; BK: butorphanol and ketamine; LBK: lidocaine, butorphanol, and ketamine; NMDA: N-methyl-D-aspartate; TT: thermal threshold

## Competing interests

The authors have no competing interests to declare.

## Authors’ contributions

JRE participated in study conception, design, coordination and execution and drafted the manuscript. SAR participated in study conception, design and coordination. RJM participated in study execution, manuscript preparation and review. BK designed and carried out the drug stability studies and drafted that portion of the manuscript. LCS participated in study conception, design, coordination and execution and helped draft the manuscript. All authors read and approved the final manuscript.

## Declarations

A portion of this work was presented in abstract form at the 2012 ACVIM Veterinary Forum.

This work was funded by the Grayson-Jockey Club Research Foundation and the Merck-Merial Summer Scholars Program. Publication costs were supported by the International Equine Colic Research Symposium.

This article has been published as part of *BMC Veterinary Research* Volume 10 Supplement 1, 2014: Selected articles from the Eleventh International Equine Colic Research Symposium. The full contents of the supplement are available online at http://www.biomedcentral.com/bmcvetres/supplements/10/S1. Publication of this supplement has been funded by The British Equine Veterinary Association.
